# Exertional Rhabdomyolysis Among U.S. Active Component Service Members, 2021–2025

**Published:** 2026-05-20

## Abstract

Exertional rhabdomyolysis is a pathologic muscle breakdown associated with strenuous physical activity. A largely preventable condition, it persists as an occupational hazard of military training and operations, especially in high heat environments among individuals pushing their endurance limits. A total of 521 cases of exertional rhabdomyolysis were identified in the U.S. Armed Forces in 2025, corresponding to a crude incidence rate of 39.7 cases per 100,000 person-years. This rate is consistent with 2023-2024 levels but remains higher than rates from 2021-2022. The percentage of inpatient cases rose to a 5-year peak of 46.3% in 2025, however, a 21.6% relative increase from the low of 38.0% recorded in 2022. In 2025, the Air Force demonstrated the most significant rate increase, 60.0%, followed by the Army, with a 16.4% increase. In contrast, the Marine Corps and Navy showed rate decreases of 32.4% and 34.8%, respectively, when compared to 2024. The Coast Guard's annual case numbers remained low, ranging 4–6 cases throughout the 5-year surveillance period. Consistent with prior reports, subgroup-specific crude rates in 2025 were highest among men, those younger than age 20 years, non-Hispanic Black service members, Marine Corps or Army members, and those in combat-specific or ‘other’ military occupations. In 2025, recruit trainees continued to experience the highest rates of exertional rhabdomyolysis, with a rate more than 6 times greater than officers and enlisted members.

**What are the new findings?**

In 2025, a total of 521 cases of exertional rhabdomyolysis resulted in a crude incidence rate of 39.7 cases per 100,000 person-years, consistent with 2023-2024 levels, yet higher than 2021-2022 levels. Meanwhile, in 2025 the proportion of cases requiring inpatient admission climbed to a 5-year peak of 46.3%, marking at 21.6% increase from the 2022 low. The period from 2024 to 2025 manifested opposite trends, with rates increasing in the Army and the Air Force while decreasing in the Marine Corps and the Navy. The Air Force showed the largest increase, with incidence rates 60% higher from the previous year.

**What is the impact on readiness and force health protection?**

Exertional rhabdomyolysis is a serious threat to military members that can limit their service effectiveness and potentially predispose them to serious injury. Risk of developing exertional rhabdomyolysis can be reduced by awareness of environmental conditions, cognizance of troop fitness levels, emphasis on graded preconditioning prior to more strenuous training, adherence to recommended work and rest ratios with appropriate hydration schedules, especially in hot, humid weather, and prompt recognition of symptoms by commanders.

Initiation of a high-intensity physical activity at unaccustomed intensity or duration, particularly under heat stress, increases the risk of exertional rhabdomyolysis. ^1^ A potentially serious condition, exertional rhabdomyolysis requires vigilance for early diagnosis and aggressive treatment to prevent serious consequences. Rhabdomyolysis is characterized by the breakdown of skeletal muscle cells and leakage of intracellular contents (e.g., myoglobin, sarcoplasmic proteins, electrolytes) into the extracellular fluid and the circulatory system. Myoglobin is toxic to the tubular cells of the kidney and can lead to renal failure.

Rhabdomyolysis severity ranges from asymptomatic or mild elevation of serum muscle enzyme levels to life-threatening emergencies, such as electrolyte imbalances, acute kidney failure, disseminated intravascular coagulation, compartment syndrome, cardiac arrhythmia, or liver dys-function. ^1 - 4^ The characteristic triad of rhabdomyolysis symptoms are muscle pain, weakness, and red-to brown-colored urine, due to high levels of myoglobin, although over half of patients do not have all of these specific symptoms. ^5^

The standard diagnostic criteria for exertional rhabdomyolysis are muscle pain, weakness, and dark urine, or elevated serum creatine kinase (CK) levels, indicating myonecrosis, usually defined as a CK level of at least 5 times the upper limit of normal, following recent exercise. ^2 , 3 , 6^

Exertional rhabdomyolysis is most commonly identified among new recruits at recruit training and combat installations, during the first 90 days of basic training, ^7 , 8^ but it can be observed in athletes accustomed to intense training, particularly when they extend themselves to the maximal limits of their physical endurance. ^9^ The condition occurs most frequently from mid-spring through early autumn at installations that support basic combat, recruit training, or major Army or Marine Corps combat units. Recruits can be exposed to environments requiring acclimatization to high heat or humidity in hotter months, while soldiers and marines in combat units often perform rigorous unit physical training, personal fitness training, and field training exercises regardless of weather conditions. A history of heat illness or prior heat stroke has also been described as significant risk factors for service members who sustained rhabdomyolysis, ^8 , 10^ revealing the potential for co-morbid conditions.

*MSMR* annually summarizes the numbers, rates, trends, risk factors, and locations of exertional heat injury occurrences including exertional rhabdomyolysis. This report includes updated surveillance data from 2021 through 2025. Additional information about the definition, causes, and prevention of exertional rhabdomyolysis can be found in previous issues of *MSMR* . ^7^

## Methods

The surveillance period ranged from January 2021 through December 2025 and included all individuals who served in the active component of the U.S. Army, Navy, Air Force, Marine Corps, Space Force, or Coast Guard. Due to small numbers, Space Force members were included in the Air Force population. All data used to determine incident exertional rhabdomyolysis diagnoses were derived from records routinely maintained in the Defense Medical Surveillance System (DMSS). These records document both ambulatory encounters and hospitalizations of active component members of the U.S. Armed Forces in fixed military and civilian (if reimbursed through the Military Health System) hospitals and clinics worldwide.

A case of exertional rhabdomyolysis was defined as an individual with International Classification of Diseases, 9th or 10th revision (ICD-9/ICD-10) diagnostic codes in any position indicating a hospitalization (i.e., inpatient) or outpatient medical encounter record with either “rhabdomyolysis” or “myoglobinuria” listed, plus a diagnosis in any position of 1 of either “volume depletion (dehydration),” “effects of heat and light,” “effects of thirst (deprivation of water),” “exhaustion due to exposure,” or “exhaustion due to excessive exertion (overexertion)” **(Table 1)** . ^11^ Each individual could be considered an incident case of exertional rhabdomyolysis only once per calendar year.

**TABLE 1.** ICD-9 / ICD-10 Diagnostic Codes Used to Define a Case of Exertional Rhabdomyolysis

Primary condition	ICD-9	ICD-10
Rhabdomyolysis	728.88	M62.82
Myoglobinuria	791.3	R82.1
Associated conditions	ICD-9	ICD-10
Volume depletion (dehydration)	276.5 *	E86.0, E86.1, E86.9
Effects of heat and light	992.0-992.9	T67.0 * -T67.9 *
Effects of thirst (deprivation of water)	994.3	T73.1 *
Exhaustion due to exposure	994.4	T73.2 *
Exhaustion due to excessive exertion (overexertion)	994.5	T73.3 *

Abbreviations: ICD-9, International Classification of Diseases, 9th Revision; ICD-10, International Classification of Diseases, 10th Revision.

* Indicates that any subsequent digit or character is included.

Cases of rhabdomyolysis associated with trauma, intoxications, and adverse drug reactions were excluded. ^12^ For health surveillance purposes, recruit trainees were identified as active component members assigned to service-specific training locations during coincident, service-specific basic training periods. Recruit trainees were considered a separate enlisted service member category in exertional rhabdomyolysis summaries by military grade.

The surveillance data reflect the most current information available at the time of analysis. Case counts are finalized over subsequent reporting cycles to incorporate data from all treatment facilities, which may be subject to routine reporting delays. Consequently, case numbers and incidence rates for a given year may be retrospectively adjusted in future reports.

## Results

**FIGURE 1.**
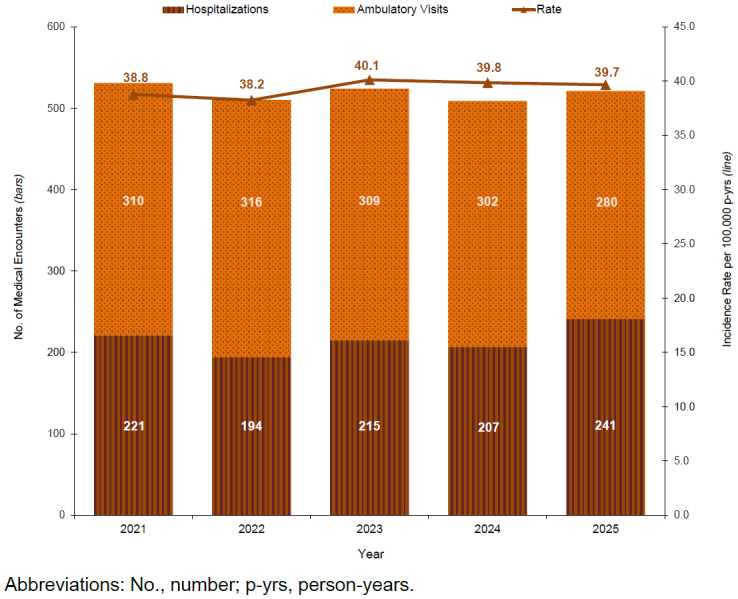
Incident Cases and Incidence Rates of Exertional Rhabdomyolysis by Source of Report and Year of Diagnosis, Active Component, U.S. Armed Forces, 2021–2025

In 2025, a total of 521 cases of rhabdomyolysis likely associated with physical exertion or heat stress (i.e., exertional rhabdomyolysis) were identified, corresponding to a crude incidence rate (IR) of 39.7 cases per 100,000 person-years (p-yrs) **(Table 2)** . This rate is consistent with 2023-2024 levels and remains higher than rates observed in 2021-2022 **(Figure 1)** . Consistent with prior annual reports, crude IRs remained highest among men, those younger than age 20 years, Marine Corps or Army members, non-Hispanic Black service members, and those in combat-specific, motor transport, and ‘other’ occupations **(Table 2)** . During the surveillance period, 2021-2025, approximately three-quarters (75.9%) of cases occurred during the warmer months (April–September) **(Figure 3)** .

**TABLE 2.** Incident Cases ^a^ and Incidence Rates ^b^ of Exertional Rhabdomyolysis, Active Component, U.S. Armed Forces, 2025

	Hospitalizations		Ambulatory Visits		Total	
	No.	Rate	No.	Rate	No.	Rate
Total	241	18.3	280	21.3	521	39.7
Sex						
Male	226	21.0	261	24.3	487	45.3
Female	15	6.3	19	8.0	34	14.2
Age, *y*						
<20	35	21.7	70	43.3	105	65.0
20–24	70	22.0	88	27.6	158	49.6
25–29	68	21.9	71	22.9	139	44.8
30–34	34	15.8	31	14.4	65	30.2
35–39	23	13.6	13	7.7	36	21.3
40+	11	7.9	7	5.0	18	13.0
Race and ethnicity						
White, non-Hispanic	101	15.4	126	19.2	227	34.6
Black, non-Hispanic	76	34.5	88	40.0	164	74.5
Hispanic	42	15.3	39	14.2	81	29.5
Other, unknown ^c^	22	13.5	27	16.6	49	30.1
Branch of service						
Army	141	31.6	166	37.2	307	68.8
Navy	15	4.5	14	4.2	29	8.7
Air Force	40	12.3	27	8.3	67	20.7
Marine Corps	44	25.9	69	40.6	113	66.4
Coast Guard	1	2.4	4	9.7	5	12.2
Military rank						
Enlisted	185	17.8	195	18.7	380	36.5
Officer	36	14.8	43	17.6	79	32.4
Recruit	20	70.6	42	148.3	62	219.0
Military occupation						
Combat-specific ^d^	53	33.2	82	51.3	135	84.5
Motor transport	11	25.4	12	27.7	23	53.1
Pilot, air crew	5	11.5	4	9.2	9	20.7
Repair, engineering	36	10.2	34	9.6	70	19.8
Communications, intelligence	47	17.5	33	12.3	80	29.8
Health care	15	14.4	13	12.4	28	26.8
Other	74	21.8	102	30.0	176	51.8
Home of record						
Midwest	38	19.6	37	19.1	75	38.6
Northeast	33	21.1	34	21.7	67	42.8
South	124	21.3	135	23.2	259	44.6
West	33	10.9	62	20.4	95	31.2
Other, unknown	13	16.7	12	15.4	25	32.2

Abbreviations: No., number; *y* , years.

a One case per person per year.

b Rate per 100,000 person-years.

c Includes those of American Indian/Alaska Native, Asian/Pacific Islander, and unknown race/ethnicity.

d Infantry/artillery/combat engineering/armor.

**FIGURE 2.**
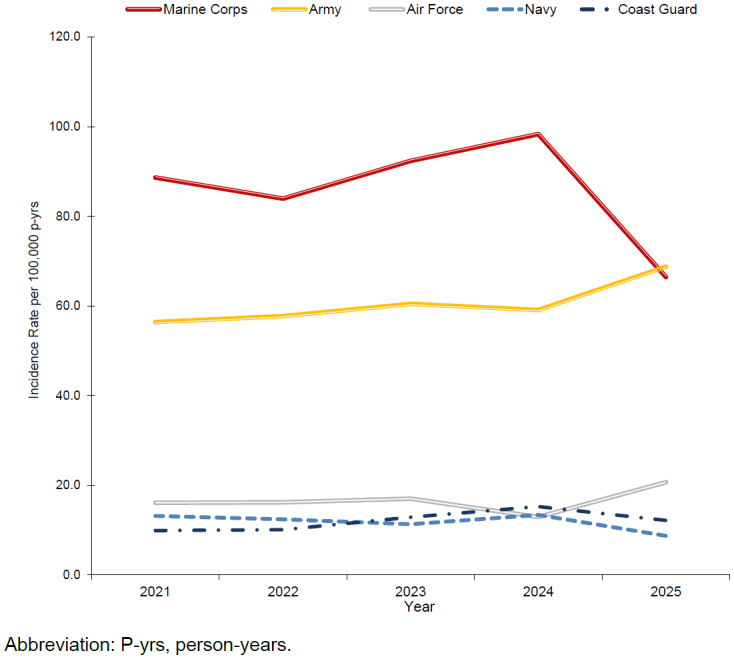
Annual Incidence Rates of Exertional Rhabdomyolysis by Service, Active Component, U.S. Armed Forces, 2021–2025

**FIGURE 3.**
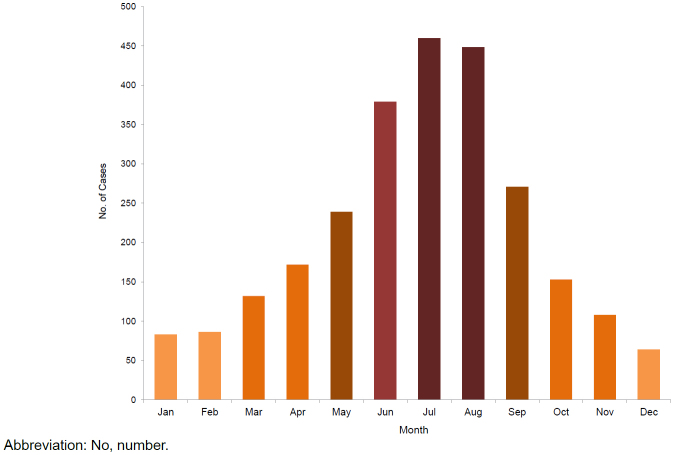
Cumulative Numbers of Exertional Rhabdomyolysis Cases by Month of Diagnosis, Active Component, U.S. Armed Forces, 2021–2025

Recruit trainees continued to have the highest rates of exertional rhabdomyolysis in 2025, at a rate of over 6 times greater than officers and enlisted members. The percentage of rhabdomyolysis cases hospitalized in 2025 was 46.3% (n=241), a 13.7% increase from 2024. Proportions of hospitalized or inpatient cases were lowest in 2022, at 38.0% **(Figure 1)** .

Over the 5-year surveillance period, the highest case counts and IRs of exertional rhabdomyolysis were observed in the Marine Corps (n=736, IR 85.9 per 100,000 p-yrs) and the Army (n=1,371, IR 60.4 per 100,000 p-yrs) (data not shown). The rates for the Air Force (n=268, IR 16.6 per 100,000 p-yrs), Navy (n=196, IR 11.8 per 100,000 p-yrs), and Coast Guard (n=24, IR 12.1 per 100,000 p-yrs) were substantially lower. The Coast Guard's annual case count remained consistently low, ranging 4–6. No cases were identified among Space Force members.

When comparing 2025 rates to 2024, the IR of the Air Force increased by 60.0%, while the IR for the Army rose by 16.4% **(Figure 2)** . In contrast, rates decreased in the Marine Corps and the Navy, by 32.4% and 34.8%, respectively. The 2025 IR of exertional rhabdomyolysis in the Navy was the lowest, at 8.7 cases per 100,000 p-yrs.

From 2024 to 2025, the IR among Hispanic service members decreased by 30.4% (from 42.4 to 29.5 cases per 100,000 p-yrs). Non-Hispanic Black service member rates were more than double those of other racial and ethnic groups, rising by 13.4% from 2024 (65.7 per 100,000 p-yrs) to 2025 (74.5 per 100,000 p-yrs). The rate remained stable for non-Hispanic White service members (34.3 in 2024 vs. 34.6 cases in 2025) (data not shown).

During the 5-year surveillance period, 22 installations diagnosed at least 20 cases each; when combined, these installations diagnosed 68.1% of all cases **(Table 3)** . Of those 22 installations, 7 support recruit or basic combat training centers: Marine Corps Recruit Depot (MCRD) Parris Island/Beaufort, South Carolina; Fort Benning, Georgia; Joint Base San Antonio-Lackland, Texas; Fort Leonard Wood, Missouri; MCRD San Diego, California; Fort Jackson, South Carolina; and Fort Sill, Oklahoma; while 11 installations support large combat troop populations: Fort Bragg, North Carolina; Fort Campbell, Kentucky; Marine Corps Base (MCB) Camp Lejeune, North Carolina; Fort Shafter, Hawai'i; Fort Hood, Texas; MCB Camp Pendleton, California; Fort Bliss, Texas; Fort Polk, Louisiana; Fort Stewart, Georgia; Fort Carson, Colorado; and Marine Corps Air Ground Combat Center (MCAGCC) Twentynine Palms, California. From 2021 through 2025, Fort Bragg, MCRD Parris Island/Beaufort, and Fort Benning together accounted for over 28.3% of all cases **(Table 3)** .

**TABLE 3.** Incident Cases of Exertional Rhabdomyolysis by Location of Diagnosis or Report (with at least 20 cases during period of surveillance), Active Component, U.S. Armed Forces, 2021–2025

Location of Diagnosis	No.	% Total
Fort Bragg, NC	298	11.5
MCRD Parris Island, SC	242	9.3
Fort Benning, GA	195	7.5
Fort Campbell, KY	119	4.6
MCB Camp Lejeune, NC	102	3.9
Fort Shafter, HI	81	3.1
Fort Hood, TX	80	3.1
MCB Camp Pendleton, CA	75	2.9
JBSA-Lackland, TX	74	2.9
Fort Leonard Wood, MO	65	2.5
MCRD San Diego, CA	59	2.3
Quantico, VA	55	2.1
Fort Bliss, TX	52	2.0
Fort Polk, LA	42	1.6
Fort Jackson, SC	37	1.4
Fort Stewart, GA	33	1.3
Fort Belvoir, VA	32	1.2
Fort Carson, CO	32	1.2
MCAGCC Twentynine Palms, CA	28	1.1
Fort Sill, OK	25	1.0
NMC Portsmouth, VA	22	0.9
Fort Gordon, GA	20	0.8
Other, unknown	827	31.9
Total	2,595	100

Abbreviations: No., number; MCRD, Marine Corps Recruit Depot; MCB, Marine Corps Base; NMC Naval Medical Center; JBSA, Joint Base San Antonio; MCAGCC, Marine Corps Air Ground Combat Center.

## Discussion

The crude IR of exertional rhabdomyolysis in 2025 was 39.7 cases per 100,000 p-yrs, consistent with 2023 and 2024 rates, remaining elevated in relation to 2021-2022 levels. While provisional data in last year's report suggested a decline in 2024, ^13^ more complete data (as of March 2026), show that the rate was consistent with those of 2023 and 2025. Overall, these data indicate a stable but elevated incidence during the latter 3 years (2023-2025) of the 5-year surveillance period compared to the initial 2 years (2021-2022).

Following last year's reporting period, divergent trends emerged, however, among the service branches: Rates increased in the Army and the Air Force and decreased in the Marine Corps and the Navy. The Air Force demonstrated the most substantial rate increase for exertional rhabdomyolysis incidence in 2025, at 60.0%, the highest among all service branches, after showing the largest decline in 2024. ^12^ In contrast, the Marine Corps and the Navy saw comparable rate decreases in 2025, of 32.4% and 34.8%, respectively, when compared to 2024 data, with the Navy recording its lowest rate in 2025.

In 2025 the percentage of cases hospitalized rose to a 5-year peak of 46.3%, a 21.6% increase from the lowest percentage, in 2022. This trend may reflect a rebound to pre-pandemic levels or increased clinical vigilance for risk mitigation that possibly led to a higher proportion of cases managed in an inpatient setting. This trend coincides with the release of the updated *Clinical Practice Guideline for the Management of Exertional Rhabdomyolysis in Warfighters* ^6^ in September 2025, which emphasizes standardized risk stratification and clearer admission criteria, focusing on early recognition of symptoms and CK thresholds.

The IR for non-Hispanic Black service members increased by 13.3% in 2025, while rates declined for Hispanic service members and remained stable for non-Hispanic White members. The persistently high IRs of exertional rhabdomyolysis among non-Hispanic Black service members (approximately twice the rates in other racial and ethnic groups), has been attributed, in part, to increased risk of exertional rhabdomyolysis among individuals with Sickle Cell Trait (SCT), ^13 - 15^ for which the carrier frequency is approximated at 1 in 13 for non-Hispanic Black individuals in the U.S. ^16 - 20^ Despite the 2023 TRADOC Regulation ^21^ formally recognizing SCT as a risk factor, with updated screening, early recognition, and prevention of exercise collapse associated with sickle cell trait (ECAST) strategies, the disparity has not improved. Further analysis into additional physiological, social, or environmental risk factors may be warranted.

The findings of this report should be interpreted with consideration of its limitations. A diagnosis of rhabdomyolysis alone does not indicate cause. Ascertaining the probable causes of exertional rhabdomyolysis cases was attempted by using a combination of ICD-9/ICD-10 diagnostic codes related to rhabdomyolysis with additional codes indicating effects of exertion, heat, or dehydration. Other ICD-9/ICD-10 codes were used to exclude cases of rhabdomyolysis that may have been secondary from trauma, intoxication, or adverse drug reactions. Recruit trainees were identified using an algorithm based on entry date into service, age, rank, location, and time in service, which was only an approximation and likely resulted in some misclassification of recruit training status.

The surveillance data for 2025 should be interpreted with caution due to reporting lags and updates of annual case counts, as data feeds are continuously uploaded and as data matures. Nevertheless, the diverging trends among the service branches from 2024 to 2025 suggest varied and possibly unequal implementation of preventive strategies within the services. These data also present an opportunity for further comparative analysis of contributing factors among the service branches. Service-specific public health and medical assets are encouraged to conduct targeted studies to identify and assess individual, operational, and environmental factors contributing to risk of exertional rhabdomyolysis, and evaluate the effectiveness of any preventive or mitigative interventions implemented.

Management after treatment for exertional rhabdomyolysis, including the decision to return to physical activity and duty, is a persistent challenge for military members and athletes. ^22 , 23^ The updated Clinical Practice Guideline provides a detailed and structured framework for return-to-duty decisions, allowing lower-risk individuals to follow a standard, criterion-based progression model, while higher-risk cases require more cautious and individualized plans, managed by specialists. The most severe consequences of exertional rhabdomyolysis are preventable with effective mitigation measures accompanied by heightened suspicion of probability when environmental conditions favor muscular injury. Commanders and supervisors at all levels should ensure that guidelines for heat illness prevention are consistently implemented, maintain vigilance for early signs of exertional heat injury, and intervene aggressively when exertional rhabdomyolysis is suspected.
